# Social ‘wanting’ dysfunction in autism: neurobiological underpinnings and treatment implications

**DOI:** 10.1186/1866-1955-4-10

**Published:** 2012-06-27

**Authors:** Gregor Kohls, Coralie Chevallier, Vanessa Troiani, Robert T Schultz

**Affiliations:** 1 Center for Autism Research, The Children's Hospital of Philadelphia, 3535 Market Street, 8th floor, Suite 860, Philadelphia, PA, 19104, USA

**Keywords:** Autism spectrum disorders, Reward, Social motivation, Ventral striatum, Ventromedial prefrontal cortex, Amygdala, Dopamine, Oxytocin, Opioids, Treatment

## Abstract

Most behavioral training regimens in autism spectrum disorders (ASD) rely on reward-based reinforcement strategies. Although proven to significantly increase both cognitive and social outcomes and successfully reduce aberrant behaviors, this approach fails to benefit a substantial number of affected individuals. Given the enormous amount of clinical and financial resources devoted to behavioral interventions, there is a surprisingly large gap in our knowledge of the basic reward mechanisms of learning in ASD. Understanding the mechanisms for reward responsiveness and reinforcement-based learning is urgently needed to better inform modifications that might improve current treatments. The fundamental goal of this review is to present a fine-grained literature analysis of reward function in ASD with reference to a validated neurobiological model of reward: the ‘wanting’/’liking’ framework. Despite some inconsistencies within the available literature, the evaluation across three converging sets of neurobiological data (neuroimaging, electrophysiological recordings, and neurochemical measures) reveals good evidence for disrupted reward-seeking tendencies in ASD, particularly in social contexts. This is most likely caused by dysfunction of the dopaminergic–oxytocinergic ‘wanting’ circuitry, including the ventral striatum, amygdala, and ventromedial prefrontal cortex. Such a conclusion is consistent with predictions derived from diagnostic criteria concerning the core social phenotype of ASD, which emphasize difficulties with spontaneous self-initiated seeking of social encounters (that is, social motivation). Existing studies suggest that social ‘wanting’ tendencies vary considerably between individuals with ASD, and that the degree of social motivation is both malleable and predictive of intervention response. Although the topic of reward responsiveness in ASD is very new, with much research still needed, the current data clearly point towards problems with incentive-based motivation and learning, with clear and important implications for treatment. Given the reliance of behavioral interventions on reinforcement-based learning principles, we believe that a systematic focus on the integrity of the reward system in ASD promises to yield many important clues, both to the underlying mechanisms causing ASD and to enhancing the efficacy of existing and new interventions.

## **Review**

### **Introduction**

Autism is currently defined by impairments in social interactions, communication and restricted interests and behaviors [1]. The core social and communicative impairments (which will probably be collapsed into one category in the forthcoming fifth edition of the *Diagnostic and Statistical Manual of Mental Disorders*) can be conceptualized as a set of related skill deficits (including social reciprocity, social perception and memory, joint attention, and perspective-taking). These deficits conspire to make it difficult for people with autism to develop and maintain social relationships [2]. Considering the symptoms of autism spectrum disorders (ASD) as developmental failure to acquire adequate social-communication skills brings into focus the learning processes that underlie ASD. Such skill-based focus has concrete implications for treatment. Currently, there are no FDA-approved medications to treat the core social and communicative skill impairments of ASD. In fact, it is probably naive to expect that a medication is by itself able to remediate a skill deficit, but it clearly might have a role in potentiating or facilitating social skill learning.

At present, most interventions targeting social-communicative skill defects and other behavioral problems in ASD rely on the principles of applied behavior analysis (ABA), especially operant techniques, where desired behaviors are reinforced using a variety of rewards (for example, verbal praise, candy, or stickers). Accumulating evidence from over 40 years of research indicates that these reinforcement-based interventions significantly increase both cognitive and social outcomes, and successfully reduce aberrant behaviors [3]. Although it is well established and has proven efficacious at the group level, this approach fails to benefit a substantial number of individuals on the autistic spectrum [4-6]. It is not yet understood how and why behavioral approaches work well for some people with ASD but not for others. As well as factors such as lack of treatment fidelity, inadequate choice of reinforcers, and absent generalization effects, reward responsiveness might be a significant moderator of intervention outcome in the context of behavior-analysis treatment programs. Reward responsiveness most likely mediates skill learning during these types of interventions [4]. Thus, the variable treatment response rate of individuals with ASD might indicate that reward systems are more efficient in those for whom behavioral interventions are most effective than in those who profit only minimally or not at all. Given the enormous amount of clinical and financial resources devoted to reinforcement-based interventions, there is a surprisingly large gap in our knowledge concerning the basic reward mechanisms in ASD. Understanding the mechanisms for reward-based learning is urgently needed to better elucidate and inform modifications to the current standard of care.

The aim of this paper was to review the biological substrates of reward processing in ASD, including neuroimaging data, electrophysiological recordings, and neurochemical measures. Because current ASD research lacks a clear reference to any validated neurobiological model of reward, we introduce a well-established framework of reward responsiveness formulated by Berridge and colleagues: the ‘wanting’/’liking’ model [7,8]. With reference to this model, we summarize what is currently known concerning the neural correlates underlying reward responsiveness in ASD, with a special emphasis on social reward versus other reward types. In this context, we discuss how the available data may not only inform the basic mechanisms of reward-based treatments in ASD, but also variability in treatment response. Ultimately, such knowledge could facilitate early diagnosis and future intervention approaches with potentially greater treatment benefits for a larger percentage of individuals with ASD. Finally, we highlight several limitations in the current ASD reward literature that probably contribute to discrepant study findings and that should be resolved in future research.

### **A heuristic of reward responsiveness: the ‘wanting’/’liking’ model**

#### **The concepts of reward ‘wanting’ and reward ‘liking’**

**Figure 1:**
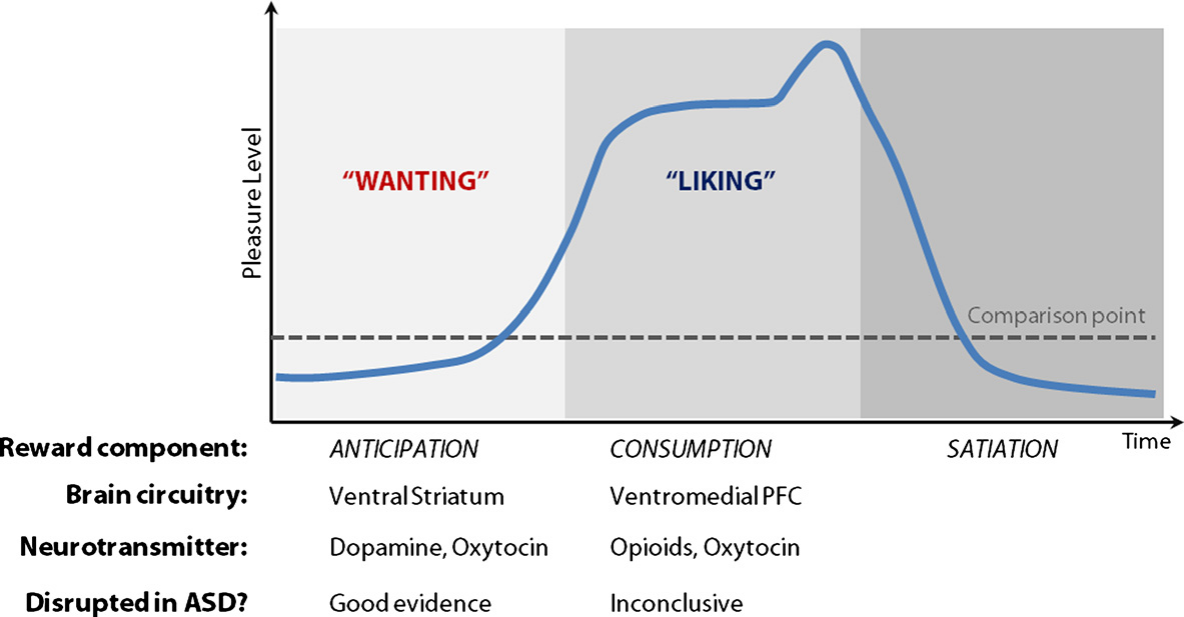
**A simplified view of the time course of reward processing and its underlying neural correlates (after Berridge and Kringelbach[**[7]**]).** Temporally, the processing of reward can be subdivided into two successive phases, with a ‘wanting’ period usually preceding a ‘liking’ response, each with a discrete neural basis. Although rewards that are ‘liked’ are typically also ‘wanted’, it seems that these two aspects of reward are dissociable both psychologically and neurobiologically. Rewarding situations are characterized by an anticipation phase or the ‘wanting’ of a reward, which often results in a phase of reward consumption or ‘liking’, with some rewards causing a peak level of subjective pleasantness (for example, a lottery win, job promotion, encounter with an old friend, favorite meal or music, sexual orgasm, drug high). Many rewarding episodes are followed by a period of satiation for the specific reward experienced. To our knowledge, there are currently no data available to suggest that the ‘wanting’/’liking’ model would apply differently to social and non-social types of reward. However, some rewards lack satiation effects or result in only short periods of satiation (for example, money). In general, physiological or drive states (for example, satiation, deprivation, stress, anxiety) strongly modulate an individual’s responsiveness to reward. Both reward ‘wanting’ and reward ‘liking’ have been associated with discrete (and to a specific extent with some overlapping) neural correlates. Whereas ‘wanting’ is mainly driven by phasic dopaminergic neural firing in the ventral striatum (including the nucleus accumbens), ‘liking’ is largely influenced by the opioid system, and recruits the ventromedial prefrontal cortex (vmPFC). As summarized in this paper, there is good evidence to suggest that reward ‘wanting’ is disrupted in ASD, particularly in the social domain, whereas the available data for reward ‘liking’ are inconclusive (see below for details).

Most people associate reward with something pleasant that they like, such as a piece of chocolate. However, hedonic feelings are only one feature of reward. Research has indeed shown that reward is not a unitary construct, but is actually comprised of different components, which can be dissociated both psychologically and neurobiologically [8]. One component is ‘liking’, which is related to the pleasurable effect of reward consumption. The other component is ‘wanting’ (also called ‘incentive salience’), which corresponds to the motivational aspect of reward; it is the anticipatory drive. Both reward components include conscious and unconscious levels of processing. On a temporal dimension, the processing of reward can be subdivided into two successive phases, with an appetitive anticipation or ‘wanting’ period usually preceding a reward consumption or ‘liking’ response (Figure 1). Typically, rewards that are ‘liked’ are also ‘wanted’. Based on learning experiences, previously neutral stimuli usually acquire reward value either through the occurrence of hedonic sensations of ‘liking’ an unconditioned stimulus (UCS) when consuming it (for example, the actual taste of chocolate) or through associations of a conditioned stimulus (CS) that predicts a reward (for example, picture of a chocolate bar). After learning, ‘wanting’ is easily triggered by encounters with an incentive CS or with a reward itself (for example, UCS). Incentive CS themselves become strongly salient, and function as motivational ‘magnets’ attracting attention, because they take on incentive properties similar to the reward they predict. This can even turn a previously neutral stimulus into an instrumental conditioned reinforcer for which people will work to obtain and ‘consume’ (for example, money). Humans possess a general intrinsic motivation system, which regulates approach behaviors towards pleasant stimuli and avoidance of threatening and stressful events. The power of this ‘wanting’ system varies from individual to individual, because of natural biological differences in reward responsiveness and learned differences in the value of different rewards.

Many rewarding episodes are followed by a period of satiation for the specific reward that was consumed. To our knowledge, there are no data available to suggest that the ‘wanting’/’liking’ model would apply differently to social and non-social types of reward. However, some rewards lack satiation effects or result in only short periods of satiation (for example, money). In general, physiological or drive states (for example, satiation, deprivation) strongly modulate an individual’s reward ‘wanting’ and ‘liking’ responses. For instance, food cues (for example, smell) are very potent in eliciting desire for food when a person is hungry, but are less salient when they have recently eaten a meal. As noted above, both reward ‘wanting’ and ‘liking’ have been associated with some distinct (and to a specific extent with some overlapping and interrelated) neural substrates, which are reviewed next.

#### **The neurobiological substrates of ‘wanting’ versus ‘liking’**

**Figure 2:**
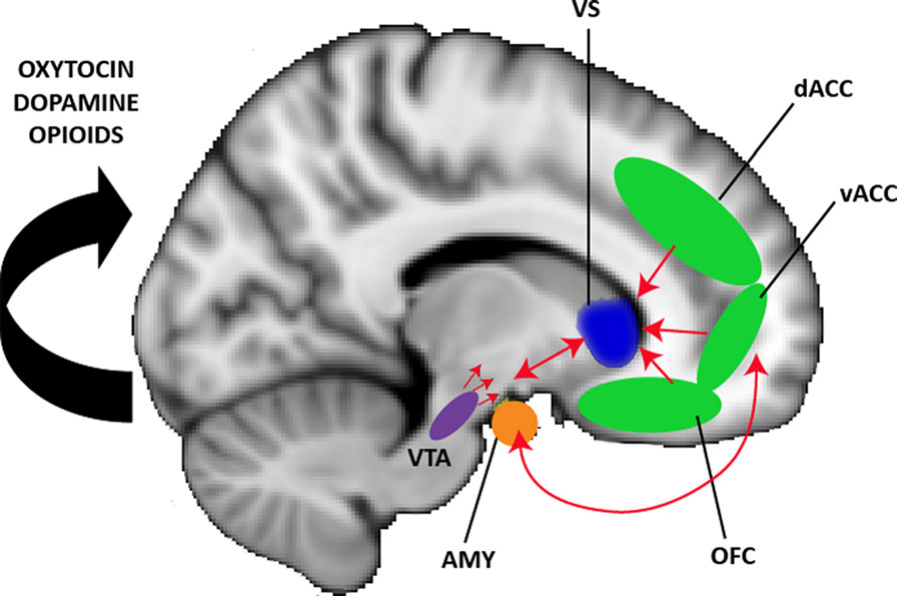
**The neural circuitry of reward ‘wanting’ versus reward ‘liking’.** The neural circuitry of reward ‘wanting’ comprises the ventral striatum (VS; blue), while that for reward ‘liking’ comprises the ventromedial prefrontal cortex, including the orbitofrontal cortex (OFC) and the dorsal and ventral anterior cingulate cortex (dACC, vACC) (green), which closely interacts with the amygdala (AMY = orange) and the midbrain, including the ventral tegmental area (VTA; purple). This complex network interfaces with motor-related areas and other higher cognitive associative cortices (not shown here) to translate basic reward information into appropriate goal-directed action plans to achieve a desired reward.

The neural circuit mediating reward-related behavior is a complex network comprising, among others, the midbrain (including the ventral tegmental area (VTA) and the substantia nigra (SN)), the amygdala, the ventral striatum (including the nucleus accumbens (NAcc)), and the ventromedial prefrontal cortex (including the medial orbitofrontal cortex (OFC) and the ventral portion of the anterior cingulate cortex (ACC)) [9] (Figure 2). Although several brain structures contribute to the reward circuitry, the central hub within this functional network is the ventral striatum (VS) [10]. The VS receives major afferent input from the OFC, the ACC, and the medial temporal lobe, including the amygdala. In addition, strong reciprocal fiber projections exist between the VS and midbrain regions. Although mostly based on anatomical research in non-human primates, recent developments in human brain imaging, such as functional connectivity measures and diffusion tensor imaging (DTI), confirm the complex information transfer within this frontolimbic network underlying reward processing [11].

Dopamine is the neurotransmitter predominately associated with reward processing [12]. Most dopaminergic neurons within the core reward circuitry, particularly in the VS, show short bursts of phasic activation in response to reward and, after learning, in response to conditioned cues that signal a potential reward [13]. Although dopamine had long been thought to mediate ‘liking’, recent evidence indicates that dopamine is neither necessary nor sufficient for generating ‘liking’ responses, but plays a more important role in the motivational component (‘wanting’) of reward [8]. More specifically, it has been suggested that the amount of phasic dopaminergic neuronal firing encodes the incentive salience of appetitive environmental stimuli, and that such firing typically precedes motor behavior to seek out, approach, and consume a reward. Animal research using *in vivo* neurochemical methods indicates that phasic dopamine signals in the VS, potentially influenced by input from the midbrain, amygdala, and ventromedial prefrontal cortex (vmPFC), underlie non-social and social reward-seeking behaviors, including eating, drinking, reproduction, and other species-specific interactions [14]. By contrast, the hedonic effect of reward is primarily associated with the opioid and endocannabinoid system [15,16].

Recent research aims to disentangle the spatiotemporal localization of both these reward-related components in the human brain using functional magnetic resonance imaging (fMRI) [17], although early fMRI studies primarily focused on money. Cued anticipation of monetary gains has been consistently found to recruit the VS, including the NAcc, with greater VS activity for more salient incentives (for example, $1 versus $5; [18]). Similarly, animal research suggests that cue-triggered VS activations precede reward consumption (for example, winning money) and primarily reflect reward ‘wanting’ . This finding has been replicated with other appetitive stimuli such as biological and social rewards [19,20], suggesting that the VS, particularly the NAcc, functions as a general, modality-independent mediator of reward ‘wanting’.

Reward ‘liking’, by contrast, has been primarily associated with activations in vmPFC, particularly the medial OFC and the ventral ACC [21]. Using prototypical fMRI paradigms designed to investigate differential brain responsiveness to reward consumption versus anticipation [18,22,23], the vmPFC has been repeatedly found to be activated during the processing of pleasant outcomes, including monetary and social rewards [24]. Insight into the neural basis of reward ‘liking’ has also been gained using pleasant-tasting food rewards. Diminished activity in the OFC has been found after a specific food item has been eaten to satiety, thereby decreasing its hedonic value and subjective pleasantness [25,26]. More specifically, a medial–lateral hedonic gradient has been indentified within the OFC, which tracks the reward value of different reinforcers with regard to its valence [27]. Medial OFC activity is related to the positive value of reinforcers (for example, winning money), whereas the lateral OFC is associated with evaluating the unpleasant aspects of reinforcement (for example, losing money). This medial–lateral gradient interacts with a second hedonic gradient along the posterior–anterior axis, which represents secondary reinforcers (such as money), more anteriorly in the OFC than primary reinforcers (such as odors, food, touch, sexual pleasure, or drugs) [15,28,29].

The ‘wanting’/’liking’ circuitry also interfaces with category-specific brain areas, allowing information about the type of reward to influence the circuit [21]; for example, social rewards such as affirmative smiles recruit reward structures and ‘social brain’ pathways [30]. This complex network interacts closely with motor-related areas and other higher cognitive associative cortices to translate basic reward information into appropriate goal-directed action plans to achieve the desired reward [9].

#### **Relevance to research into autism spectrum disorders**

Although the human fMRI literature is arguably more complex than the simple VS (‘wanting’) versus vmPFC (‘liking’) dichotomy described above [31], this framework provides a useful heuristic model to evaluate reward responsiveness in individuals with ASD. To date, little is known about reward function in ASD, and conflicting evidence comes from intervention programs versus experimental research.

On the one hand, behavior analytic intervention programs, which place reward-based reinforcement at the heart of their treatment system, have been repeatedly found to improve socially appropriate behavior and cognitive skills while diminishing dysfunctional activities [32]. Reward-based interventions draw on a variety of reinforcers (food, tokens, sensory stimulation, toys, idiosyncratic preferred objects, praise [33]), which act as key levers for learning. For instance, when a positive reinforcer follows a desired behavior, the future frequency of that behavior is enhanced under similar conditions. By contrast, when positive punishment (for example, disapproval) follows an undesired behavior, the future frequency of that behavior is decreased under similar conditions. On the other hand, evidence from behavioral experiments suggests that individuals with ASD have diminished responsiveness to reward. Stimulus–reward association learning has been repeatedly highlighted as an area of difficulty for children with ASD [34,35], and variability in reward-learning skills has been identified as an important predictor of social-communication abilities [36]. Interestingly, the deficit in reward learning (and its link to social skills) seems to persist through to adulthood, as evidenced by impairments in the rapid formation of reward–stimulus associations and its correlation with clinical symptoms of social dysfunction [37-39].

Furthermore, both intervention research and behavioral investigations have suggested that individuals with ASD might be characterized by particularly low responsiveness to social rewards such as facial expressions (for example, smile), spoken language (for example, praise), and gestures (for example, the thumbs-up gesture) [40,41]. In fact, in behavioral treatment programs, young children with ASD profit less from the use of social rewards than from non-social reinforcers [42,43], and several experimental studies have confirmed that, relative to typically developing children (TDC), the performance of children with ASD is only minimally affected by social reinforcement [44-47].

To date, the paradoxical finding of efficacious treatments rooted in reinforcement strategies in combination with weaker reward systems in ASD has received little attention in the field. This highlights a gap in our understanding of the underlying cognitive and biological processes that contribute to treatment response. In particular, a potentially important limitation of current experimental and intervention research in ASD is that it tends to construe reward as a unitary phenomenon, lacking a clear reference to any validated neurobiological model of reward; however, a critical examination of reward function in ASD requires a more fine-grained analytic approach. For instance, lower responsiveness to social reward as evident at the behavioral level could be the result of diminished ‘wanting’ or ‘liking’, or both. More specifically, reward ‘liking’ usually triggers and directs reward ‘wanting’ so that the extent to which a reward is wanted typically depends on the degree to which it has been liked [7]. However, in some psychiatric disorders, such as addiction, schizophrenia, and depression, ‘wanting’ and ‘liking’ can become uncoupled as a result of circumscribed neurobiological dysfunctions [48]. For example, a disruption in dopamine function might cause diminished ‘wanting’ and approach behavior to obtain a specific rewarding stimulus, even if the ‘liking’ response to that particular reward is preserved. In the case of schizophrenia, anhedonia (the reduced capacity to experience pleasure or ‘liking’), has long been considered to be a cardinal symptom of patients with this disorder [49]. However, recent studies using a range of pleasant stimuli, including positive words, faces, sounds, film clips, erotic pictures, and sweet drinks, have highlighted that the ability to experience pleasure is generally intact in individuals with schizophrenia, whereas the capacity to pursue and achieve a pleasurable goal (that is, the ‘wanting’ component of reward), is significantly disrupted [50]. Several authoritative reviews thus concluded that anhedonia (diminished ‘liking’) is a less prominent feature of schizophrenia than avolition (diminished ‘wanting’) [49,51-53].

This example clearly illustrates that consulting the ‘wanting’/’liking’ model is particularly helpful to identify which aspect of reward function is compromised or preserved in different psychopathologies. Such information might facilitate efforts at early identification and could have important implications for prevention and intervention programs. In the case of ASD, an improved understanding of distinct reward functions and their respective disruption may help to isolate discrete reward subprocesses (‘wanting’ versus ‘liking’) and their associated biological substrates (VS versus vmPFC) as treatment targets.

Given that there are currently no objective behavioral markers of ‘liking’ and ‘wanting’, it is necessary to draw on neurobiological measures. Three sets of data are considered in this review: 1) functional neuroimaging signals, 2) electrophysiological recordings, and 3) neurochemical data. Several preliminary predictions can be made with respect to the ‘wanting’/’liking’ model. If ‘wanting’ is compromised in ASD we would expect to see 1) aberrant brain responses in the VS, 2) atypical event-related brain potentials (ERPs) and EEG patterns associated with the anticipatory aspect of reward, and (3) disrupted dopamine function. On the other hand, if ‘liking’ is negatively affected, we would predict 1) aberrant brain activation in the vmPFC, 2) atypical ERP and EEG responses related to reward outcome processing, and 3) disrupted opioid function. Considering the core social phenotype of ASD (for example, ‘lack of spontaneous seeking to share enjoyment, interests, or achievements with other people’ [1]), it can be speculated that both ‘wanting’ and ‘liking’ of social reward is compromised in this disorder, with the most pronounced disruptions to be expected for social reward ‘wanting’ (that is, social motivation). In the following sections, we evaluate the extent to which the proposed predictions are supported by the available data.

### **Reward responsiveness at the neurobiological level in ASD**

#### **Functional magnetic resonance imaging**

Although the involvement of the mesocorticolimbic reward circuitry in the psychopathology of ASD has been discussed in the literature for many years [40,41,54-58], only recently has research begun to systematically evaluate potential malfunctions within this circuitry. In the following section, we review the handful of studies that used fMRI to compare the blood oxygen level-dependent (BOLD) signal in response to different types of reward in children and adults with ASD relative to typically developing controls. There are complex sets of data reported across the different studies, but in this paper, we focus exclusively on the VS and the vmPFC as the neural substrates of reward ‘wanting and ‘liking’ respectively. Further, because the amygdala forms a unique microcircuitry with the VS and the vmPFC to promote reward-seeking behaviors [59], and has been repeatedly suggested to be dysfunctional in ASD [41], we also review the amygdala-related findings in more detail.

#### ***The ventral striatum and reward ‘wanting’***

The available data suggest that ‘wanting’ (the motivational drive to achieve reward) is compromised in ASD. Four out of five published fMRI studies reported diminished VS activation in individuals with ASD compared with TDC when processing either social or monetary reward versus non-reward [30,60-62]. In two studies, Dichter and colleagues compared neural activation in samples of adults with and without ASD during a delayed anticipation task with two different reward contingencies. First, they tested brain responses to money and typical autism-specific objects of interest (for example, trains, cars, plastic bricks) and found decreased VS activation in ASD during periods of money anticipation and outcome, whereas VS activity was present for typical autism-specific objects of interest [60]. In a follow-up study applying the same paradigm but with a focus on social (for example, faces) versus monetary reward, adults with ASD again showed lower brain activation in the VS during money anticipation, but did not reveal VS hypoactivation for face rewards [61]. An early study by Schmitz and colleagues applied a monetarily rewarded sustained attention task to adults with and without ASD, but did not report VS activation in either group [63]. Scott-Van Zeeland and colleagues [62] were the first to compare BOLD responses to both monetary and social reward (for example, smiling face combined with verbal praise) in children with and without ASD performing an implicit learning task. In this study, the ASD group displayed diminished activation in the VS for social reward, but not for monetary reward. In addition, VS activation to social reward predicted social capacities (as measured by the Social Responsiveness Scale) within the TDC group, but not the ASD group. Kohls *et al*. [30] also tested children with and without ASD, and investigated BOLD responses to social and monetary reward in the context of an incentive go/no-go paradigm. Similar to the stimuli by Scott-Van Zeeland and colleagues [62], approving faces that were contingent on accurate task performance were used as social reinforcers. Despite normal reward responsiveness at the behavioral level, participants with ASD showed hypoactivation in the VS under monetary reward conditions that required an active response to obtain a reward. Contrary to the authors’ predictions and to the results of the previous study [62], significantly reduced VS responses during social reward processing were not seen, but these findings are consistent with results from Dichter *et al*. [61].

Taken together, blunted VS activity is a replicated phenomenon in children and adults with ASD, and might represent a neurobiological marker for diminished incentive salience (‘wanting’) related to social and/or monetary reward. Compromised ‘wanting’ possibly disrupts the tendency in ASD to self-initiate goal-directed actions to seek out specific environmental rewards (for example, social incentives), whereas motivational tendencies towards strongly preferred idiosyncratic rewards seem to be preserved; typical autism-specific objects of interest led to normal VS activation suggestive of intact ‘wanting’ for this type of incentive. However, it should be acknowledged that the reviewed data provide a somewhat inconsistent picture about the specificity of VS disruption to social versus monetary reward. It is beyond the scope of this paper to speculate upon the diverse subject- and method-related factors that might have contributed to these inconsistencies (for a thorough discussion, see Kohls *et al*. [30]). Importantly, however, although monetary reinforcers have predominantly been operationalized and used as non-social stimuli, money is imbued with social connotations and exerts a substantial influence on pro-social behavior [64-66]. In this regard, aberrant VS responses to monetary incentives would not necessarily be at odds with the autism social phenotype. In addition, different potencies of social reward have been applied across studies, which could explain the discrepant results with respect to this type of reward. A picture of a smiling face paired with verbal praise was used as social reinforcement by Scott-Van Zeeland *et al*., whereas Dichter *et al*. and Kohls *et al*. chose static face rewards without praise. It seems likely that the combination of facial rewards with praise may represent a stronger social incentive with correspondingly greater reward system responsiveness, primarily in TDC, making it more probably that activation differences are detected between individuals with and without ASD within the VS. Future research should address these issues.

#### ***The ventromedial prefrontal cortex and reward ‘liking’***

Regarding the vmPFC as the mediator of reward valuation or ‘liking’, the available imaging data are rather mixed. For the vmPFC (including rostral–ventral ACC and medial OFC), two studies reported stronger activation [62,63] and two reported lower activation [30,61] in ASD compared with TDC in response to monetary reward. In Schmitz’s study [63], ventral ACC activity correlated positively with social symptom severity (ADI-R), suggesting a possible link between atypical reward consumption and social functioning. Another study showed diminished activation in the vmPFC under social reward conditions [30], which is in contrast to data from Dichter *et al*. [61] and Scott-Van Zeeland *et al*. [62]. Lastly, one investigation found greater activation in the vmPFC in response to autism-specific objects of interest in individuals with ASD relative to typical control participants [60].

In summary, the current ASD imaging literature presents no clear pattern of results with respect to possible differences from controls for reward consumption or ‘liking’. Interestingly, however, enhanced activation in the vmPFC in response to high autism-interest objects suggests that the hedonic value of such objects is greater in individuals with ASD than in TDC. This idea is in line with literature showing that certain classes of objects and topics, which often constitute circumscribed interests, are perceived as pleasurable by many affected individuals [67], and the use of such items in behavior-analysis intervention programs has been found to be therapeutically effective [68,69]. However, on a day-to-day basis, these strongly ‘liked’ circumscribed interests are likely to interfere with social functioning.

#### ***The amygdala as a salience detector***

The amygdala is thought to influence and amplify the perception of emotionally and motivationally potent stimuli at very early stages in their processing. It tracks relevant positive and negative events in the environment and contributes to appropriate adaptation of behavior (for example, approach or avoidance reactions [70]). Additionally, amygdala function is crucial for making an association between a specific stimulus (for example, face of an unknown person) and the affective experiences intrinsically associated with this stimulus (for example, pleasant social interaction with this person), linking initially neutral environmental stimuli with motivational significance [71].

The amygdala has been repeatedly linked to the social deficits present in ASD [41,56]. For instance, in an interesting fMRI study, Grelotti and colleagues [72] found weaker amygdala activation for faces than for cartoon characters (for example, Digimon ‘Digital Monsters’) in an autistic boy with a strong preoccupation with these characters, whereas a matched typical control boy showed the expected opposite neural activation pattern. The strong amygdala engagement with the cartoon characters seemed to reflect the exaggerated motivational salience tagged to this idiosyncratic interest relative to faces. Put another way, decreased amygdala activation for faces might reflect a lack of proper appetitive value assigned to this class of stimuli [41,73].

The study by Dichter and colleagues [61] on reward processing revealed hyperactivation in the amygdala in adult participants with ASD while they were anticipating social reward. This activation correlated positively with social symptom severity (Autism Diagnostic Observation Schedule-Generic ADOS-G). By contrast, Kohls and co-authors [30] found hypoactivation in this brain area under social reward conditions in children with the disorder. Both studies used very similar experimental task designs with comparable reward contingencies. The inconsistent finding might be due to the different ages studied in the two papers, as other data suggest that there could be an abnormal developmental trajectory of amygdala reactivity to social incentives in ASD [74,75]. Systematic research is clearly needed to address this idea and its implications for the development of aberrant socially motivated behavior in ASD.

#### ***Synopsis***

In summary, the vmPFC–VS–amygdala circuitry seems to be dysfunctional in ASD, and to form, at least partially, the basis for atypical reward responsiveness in individuals with ASD. Preliminary evidence indicates that the motivational component of reward (the ‘wanting’) might be particularly compromised in individuals with ASD. This is reflected in blunted VS activity, which, however, seems to be dependent on the incentive at stake (that is, low versus high autism-interest rewards).

Dysfunction within the vmPFC–VS–amygdala system, such as an insufficient communication between the amygdala and/or the vmPFC to the VS, has been proposed to underlie aberrant motivation to seek out detrimental substances at the expense of ‘natural’ rewards in other psychopathologies (for example, addiction [76,77]). It can therefore be hypothesized that an atypical pattern of brain activity within this circuitry in individuals with ASD may trigger strong seeking of salient, autism-specific rewards at the cost of neglecting other essential environmental rewards, including social rewards. In fact, several recent imaging studies on resting-state functional connectivity and DTI confirm disruptive neural activation dynamics in ASD within the vmPFC–VS–amygdala circuitry [78-81]. These findings are also in line with the idea of ASD as a neurofunctional disconnection syndrome [82-84], most likely mediated by complex genetic factors (for example, synaptic cell adhesion plasticity [85]), which affect efficient information transfer within the mesocorticolimbic reward circuitry and may cause aberrant motivation, that is, affect ‘wanting’ tendencies.

### **Event-related brain potentials and resting-state EEG**

Despite the fine spatial resolution of functional MRI, one major limitation is its restricted temporal precision. For instance, the BOLD signal in the VS evoked by reward-predicting cues has been shown to rise at 2 seconds, to peak between 4 and 6 seconds, and to fall back to baseline after 10 to 12 seconds [86]. In contrast to the relative slowness of the brain’s BOLD response as measured by fMRI, electrophysiological recordings such as electroencephalography (EEG) and ERP provide measures with exquisite real-time temporal resolution on the scale of milliseconds [87]. Thus, EEG and ERP might be specifically suited to address the question about the extent to which temporal phase of reward processing might be compromised in ASD (reward anticipation/’wanting’ versus reward consumption/‘liking’). In the next section, we summarize the current knowledge with regard to electrophysiological correlates underlying reward responsiveness in individuals with ASD relative to controls.

#### ***Event-related brain potentials components related to ‘wanting’ and ‘liking’***

Two ERP components are especially relevant to the ‘wanting’/’liking’ framework: the feedback-related negativity (FRN) and the P3 component. Although these two ERP correlates are associated with well-described functional roles in the cognitive neuroscience literature (FRN with external reward monitoring; P3 with selective attention allocation), both have been repeatedly described as indirect neural indices of reward responsiveness. The P3 and the FRN can be elicited by reward-predicting cues and reward outcome. However, research and theory suggests that the P3 is more closely related to reward-seeking behaviors (‘wanting’) and the FRN to reward consumption (‘liking’ or ’disliking’) [88,89].

The P3 is a positive ERP component with a maximum deflection at parietocentral electrodes (for example, Pz), whereas the FRN is a negative deflection, which has its largest amplitudes at frontocentral sites (for example, FCz). Each component peaks around 300 ms after the onset of a critical stimulus. However, whereas the P3 has been found to be sensitive to reward magnitude (that is, larger amplitudes for high versus low reward) and reward valence (that is, larger amplitudes for reward gain versus loss), the FRN is modulated almost exclusively by reward valence, with more negative waveforms in response to non-reward outcome relative to reward gain [90]. Moreover, both components are influenced by an individual’s task engagement, so that larger amplitudes result from active goal-directed responding to achieve a reward compared with the passive receipt of a reward [91]. Although most normative studies have focused on the effect of monetary reward on these components, more recently, two reports showed that social rewards (for example, affirmative faces) elicited robust P3 and FRN responses comparable with those evoked by monetary rewards [92,93]. Additionally, different personality dimensions, including reward dependence, seem to determine the extent to which both waveforms are modulated by reward in the normal population [94,95].

According to the locus coeruleus norepinephrine (LC-NE) P3 hypothesis, the P3 component reflects a short, phasic signal of the widely distributed and synchronously active LC-NE system, which closely interacts with the reward circuitry (for example, vmPFC, amygdala) to evaluate the salience of an incoming stimulus and, as a result, to optimize active reward-seeking (‘wanting’) behaviors [89]. By contrast, the FRN can be understood as a general manifestation of a reward-monitoring system that recognizes discrepancies of outcome expectancies during reward consumption, for example, if a ‘liked’ reward is expected but not delivered, it elicits a ‘disliking’ signal, which is reflected in a negative ERP response. Such a mechanism enables an individual to adjust their behavior adequately so that the reward benefit can be maximized in the future. The vmPFC (that is, ACC) and the striatum have both been suggested as potential sources for the scalp-recorded FRN response [96-98]; however, the involvement of the striatum is less likely [99].

#### ***Feedback-related negativity, P3, and reward responsiveness***

The field of ASD has a long and rich tradition of using ERP measures to acquire detailed real-time information about the dynamics and integrity of neural processes in the brain of individuals with ASD [100]. However, research has just started to evaluate the clinical utility of the P3 and the FRN as potential markers for abnormal reward responsiveness in ASD. In the following sections, we present recent relevant findings and interpret them in the framework of reward anticipation (’wanting’) versus reward consumption (’liking’).

Groen and colleagues [101] investigated ERP responses in a mildly impaired group of children with pervasive developmental disorder not otherwise specified (PDD-NOS) while they performed a reinforcement-based learning task with performance feedback (winning or losing points). There was a robust P3 effect in response to feedback outcome. A P3 related to feedback anticipation was not reported. The participants with PDD-NOS did not differ from a TDC group in their outcome-evoked P3, suggesting that feedback processing was intact in this patient group. Interestingly, however, during the anticipation of positive feedback, the PDD-NOS group displayed an atypical stimulus-preceding negativity (SPN), an ERP component that is thought to index reward anticipation, similar to the P3 [102].

Larson and colleagues [103] used a gambling task to specifically elicit the FRN and P3 in response to monetary gain versus loss in children with and without ASD. Reward anticipation was not assessed in this study. Similar to the findings by Groen [101], the ERPs evoked by reward outcome did not differ between the experimental groups. The authors concluded that the neural response to concrete, external feedback, that is, monetary gain (‘liking’) and loss (‘disliking’), is intact in ASD, reflected in normal FRN and P3 effects.

It should be noted that both Groen and Larson only used one type of incentive in their studies, points and money respectively, which leaves unclear the extent to which their findings may also be relevant for other fundamental types of appetitive stimuli such as social rewards. Kohls and colleagues [92] were the first to compare the effect of social (that is, affirmative faces) and monetary incentives on ERP responses in children with ASD versus TDC. They adopted a cued go/no-go paradigm from the animal literature, which has been widely used to assess reward anticipation (initiated by cue signals) followed by goal-directed behavior (for example, button press or inhibitory response) and a potential rewarding outcome [104]. The authors focused on the P3 as the ERP component of interest; the task design was not suited to evoke the FRN. Consistent with the findings of Groen *et al*. [102] and Larson *et al*. [103], the outcome-related P3 did not differentiate between ASD and TDC participants. However, whereas the TDC group exhibited an increased P3 in response to cues that signaled a potential social or monetary reward, relative to non-reward, the ASD group did not show this enhancement effect, and even showed diminished P3 activity in response to cues that triggered a phase of social reward anticipation. Moreover, P3 activity elicited by incentive cues in both social and monetary reward conditions correlated negatively with social symptom severity (ADOS-G), suggesting that children with ASD who had stronger social deficits had weaker modulation of the go-cue P3 when reward was at stake. Based on the LC-NE P3 theory, the authors concluded that the ERP data indicate an attenuated state of motivated attention allocation, particularly towards signals that trigger active reward-seeking (‘wanting’) behavior in individuals with ASD [105].

Although it is premature to draw conclusions from only three ERP reports, the evidence suggests that outcome-related neural responses are less impaired in ASD (reflective of relatively intact ‘liking’) than are brain potentials related to the anticipatory period preceding reward consumption (reflective of disrupted ‘wanting’), based on the incentives used to date. This neural dysfunction involves both social and non-social (for example, monetary) reward, with a more pronounced deficit for social incentives.

#### ***Frontal alpha power asymmetries***

The strength of reward approach tendencies can be assessed across the age spectrum with active- and resting-state EEG by calculating hemispheric alpha power asymmetries over the frontal cortex [106]. Individuals with greater frontal alpha activity on the left relative to the right hemisphere display more reward-seeking behaviors than do individuals with greater activity on the right side. The left vmPFC has been suggested as the potential source for stronger left-sided alpha-band activity [107]. Owing to the relatively limited spatial resolution of EEG source localization techniques, it is not yet clear to what extent other reward structures contribute to the scalp-recorded alpha asymmetries. Because of its involvement in reward ‘wanting’, one likely candidate is the dopaminergic VS [108,109].

With regard to autism, Sutton and colleagues [110] were the first to investigate the relationship between resting-state frontal alpha asymmetry and symptom severity expression in ASD. Children with ASD who showed left frontal EEG asymmetry were reported by their parents to have fewer symptoms of social impairment compared with children with right frontal asymmetry; however, the former was accompanied by greater levels of social anxiety and stress. These findings suggest that children with ASD with left frontal asymmetry might be more motivated to participate in social interactions, possibly because of stronger ‘wanting’ tendencies. A stronger inclination to seek out social interactions may make the appearance of social impairments less severe, resulting in reduced reports of symptoms, whereas, the motivation to interact with others, coupled with an underdeveloped behavioral repertoire to do so, might result in heightened levels of social stress and anxiety [111]. Interestingly, the left asymmetry subgroup of children with ASD has a great resemblance to the ‘active-but-odd’ clinical subtype described by Wing and Gould [112], whereas the right asymmetry group is more consistent with the ‘passive’ or ‘aloof’ subtypes [111]. Dawson and colleagues [113] first noted differences in frontal alpha power in children with ASD classified as ‘active-but-odd’ versus ‘passive’. This was replicated recently by Burnette and colleagues [114], who also found that left frontal alpha asymmetry during resting state was associated with later age of onset of ASD-specific symptoms based on parental report. This could indicate that greater social interest (‘wanting’) may obscure social symptom presentation in young children, resulting in delayed identification.

In a first attempt to measure frontal alpha activity during an active task, Kylliäinen and colleagues [115] recently reported relatively greater left-sided frontal alpha activity in TDC during viewing of faces with direct eye gaze, reflective of motivational social approach [116], a pattern that was absent in children with ASD. By contrast, no group differences were detected in frontal alpha responses to non-social control stimuli, such as automobiles. The authors concluded that social attention as expressed by direct eye contact may not be socially rewarding for children with ASD, and thus, does not properly activate their approach-related brain mechanisms. Interestingly, the data did not support the assumption of greater aversion-related physiological responses to direct eye gaze in ASD relative to controls that have been made in previous reports [117,118]. However, it should be noted that left frontal alpha asymmetries have not been linked exclusively to reward seeking in the social domain. Stronger responsiveness to monetary incentives also correlates with larger left frontal alpha power in typical individuals [107], indicating that EEG asymmetries reflect more general motivation tendencies rather than specifically social ones. With regard to ASD, further studies are warranted that assess, for instance, frontal alpha activity in response to stimuli of high autism-specific interest to test the extent to which an exaggerated reward value of this type of stimuli contributes to the presentation of the autism social phenotype (for example, interference with socially motivated approach behaviors).

#### ***Synopsis***

In summary, electrophysiological studies in ASD show atypical results related to the anticipatory (or ‘wanting’) aspect of rewarding events, especially in the social domain, which may affect social orienting and approach. However, the degree to which this impairment reflects an intrinsic versus a learned process (or a combination of both), and its specificity to social stimuli (versus, for example, autism-specific objects of interest), is as yet unresolved. The literature suggests that stronger social interest can potentially overshadow symptom expression in young children with ASD, preventing early diagnosis. Therefore, information about social motivation profiles acquired through either behavioral and/or electrophysiological measures (for example, frontal alpha asymmetry) is crucial to facilitate efforts at early identification. Moreover, future inquiries will reveal the extent to which EEG investigations can assist in determining which children with ASD are likely to be treatment responders and which might require special or modified treatment efforts.

### **Neurotransmitters and neuropeptides**

Although multiple reward-related neurotransmitters and neuropeptides have been implicated as atypical in ASD, we focus here on dopamine, opioid, and oxytocin (dys)function, because of their potential effect on reward ‘wanting’ and ‘liking’ [56]. Dopamine and endogenous opioids are well-known neurochemicals with circumscribed roles in motivational behavior such as reward-seeking versus consumption, respectively [119], whereas the peptide oxytocin has only recently become established as a facilitator of reward signaling and learning, particularly in social contexts [120]. Thus, all three molecules provide independent contributions to rewarding effects, as described below.

#### ***The dopaminergic ‘wanting’ system***

Dopamine mediates a variety of behaviors and functions, including selective attention, learning, motor functioning, hormone release, and goal-directed motivated behaviors [121]. In this context, dopamine primarily encodes the incentive value (or the ‘wanting’) of reward. It is released in response to contextual cues predictive of reward, initiating a phase of reward anticipation and approach. Animal research shows that dopamine antagonists or agonists injected into the VTA and VS impair or facilitate, respectively, reward approach behaviors, but not reward consumption [119]. In humans, drug-induced activity in the VS is linked to feelings of craving and ‘wanting’, but not to feelings of euphoria or pleasure [122].

Dysfunction in the dopamine system in ASD has been suggested, based on the beneficial effects of dopamine receptor antagonists (for example, antipsychotic drugs such as risperidone) in treating certain symptoms commonly exhibited by affected individuals, such as stereotypies, aggression, hyperactivity, and self-injury [123]. Because such symptoms can be induced in animals by increasing the dopamine level, it has been inferred that ASD might be associated with mesocorticolimbic dopaminergic overactivity. However, conflicting results have been found in studies measuring peripheral (for example, blood, urine) or central (for example, cerebrospinal fluid (CSF)) levels of dopamine and its metabolites, with some studies reporting atypical dopamine turnover in patients [121]. Evidence is also scarce and inconclusive with regard to dopamine-related neuroimaging using positron emission tomography (PET) or single photon emission computed tomography (SPECT) in individuals with ASD. For instance, Ernst and colleagues [124] found reduced dopamine metabolism in the vmPFC, but not in the VS, in children with ASD compared with controls. However, follow-up studies could not confirm this early finding, and reported either enhanced dopamine bindings in the vmPFC [125], in the VS [126,127], or in both brain areas [128], or did not find any abnormalities in ASD [129]. It should be noted that urine, blood, CSF and baseline PET/SPECT measurements usually assess stable, tonic dopamine levels, whereas the beneficial effects of antipsychotic drugs stem from blocking phasic dopamine release, which only minimally contributes to these tonic levels [130]. This raises the possibility of a dysfunction in the phasic rather than the tonic dopamine metabolism in ASD, which would be more consistent with the neuroimaging and electrophysiological findings of atypical reward ‘wanting’. Indeed, reward-predicting signals and behaviorally important events (for example, novel stimuli) elicit brief, phasic, bursts of dopamine impulses, which last less than 500 ms, and prompt reward anticipation [12]. Research provides evidence that specific subpopulations of dopaminergic cells within the VS respond differently depending on reinforcer type. It has been shown that some striatal cell groups encode primary reinforcers (for example, water, food, sexual intercourse), whereas others are thought to be ‘idle’ and modifiable through reward-based learning (for example, drug conditioning [131]). The idea that dopaminergic cell activity tracks different reward types is intriguing, because it might offer a simplistic, although plausible, explanation as to why some incentives (for example, objects of circumscribed interest) induce goal-directed approach behavior in individuals with ASD, whereas others (for example, social reward) do not. Such ‘selective’ impairment could be thought of as genetically driven [132,133], or acquired through aberrant learning experiences, or both. Because single-cell recordings are mostly limited to animal research, the use of mouse models of ASD could be a fruitful approach to test the merits of this idea [134].

#### ***The opioid ‘liking’ system***

Behavioral effects of opiate administration include symptoms such as insensitivity to pain, social withdrawal, motor hyperactivity, repetitive and stereotyped behaviors, and hypersensitivity to sensory stimulation. This overlap with ASD symptoms has led to the idea of an opioid dysfunction in autism [135-140]. Endogenous opioids (or opiates, such as beta-endorphins or enkephalins) are peptides that act as neuromodulators in the CNS and dock at receptors activated by morphine, an alkaloid extracted from opium, and related substances [141]. Thus, the euphoric and narcotic effects elicited by morphine are thought to be shared by endogenous opioids produced by the body. A large body of evidence from animal and human research suggests a role of endogenous opioids in pleasant social and non-social behaviors, including sexual activity, social interactions, play, grooming, and food intake. In humans, endogenous opioids increase subjective feelings of interpersonal warmth, euphoria, and calmness, but decrease incentive motivation. It has been proposed that endogenous opioids induce pleasure and bring consummatory behaviors to a satisfying conclusion [119,142,143]. This ‘liking’ role is emphasized by the fact that the vmPFC, a brain area that is explicitly associated with reward ‘liking’, contains a particularly high density of opioid receptors [15].

The data on the possible role of opioid dysfunction in ASD has been conflicting, with opioid measurements from urine, blood plasma and CSF being reported as decreased, increased or normal [121]. Some authors argue that there might be subtle alterations in opioid functioning in ASD (for example, increased C-terminally directed beta-endorphin protein immunoreactivity, but normal N-terminally directed activity), whereas others suggest that such aberrant opioid levels are not specific to ASD [56]. Moreover, because of the putative role of endogenous opioids in the regulation of social behavior, several investigations have examined the effect of opioid receptor antagonists (for example, naltrexone) on symptom expression in ASD. Despite some modest effects on maladaptive behaviors, for example, irritability, hyperactivity, self-injury, most controlled studies suggest that the efficacy of this treatment is limited, especially with regard to the social symptoms of ASD [144]. Taken together, there is no consistent evidence that atypical opioid functioning is a determining factor for the core phenotype of ASD. Considering that endogenous opioids mediate the hedonic aspect of reward, this conclusion is in line with the neurophysiological findings that reward ‘liking’ is less compromised than ‘wanting’ in affected individuals.

#### ***The oxytocin system as neuromodulator of ‘wanting’ and ‘liking’***

Several lines of inquiry suggest that social cognition and behavior are regulated by a combination of dopamine, opioids and the hormone oxytocin [145,146]. Oxytocin is a nine amino-acid peptide, which is synthesized in the paraventricular and supraoptic nucleus of the hypothalamus, and released into the bloodstream by the posterior pituitary with wide distribution in the central nervous system [147,148]. Oxytocin is best known for its contribution to numerous social functions in humans and animals, including social recognition, species-specific interactions, attachment, and other pro-social behavior [120,149-151]. Cumulative evidence is consistent with the view that oxytocin enhances the motivation for social interactions through a complex functional system. This involves increased social attention and memory and reduced social stress and social anxiety, which in combination promote the ability and willingness of an individual to repeatedly take risks in approach, cooperative, and trusting behaviors [152]. Oxytocin exerts its effects on social motivation through a variety of neurochemicals among which dopamine and opioids are key players. Both dopamine (‘wanting’) and opioids (‘liking’) mediate social encounters and eventually the formation of pleasant social rewards and memories associated with such occurrences. In turn, this increases the likelihood of an individual to seek out these stimuli in the future [119].

External contextual cues predictive of potential social reward (for example, face or voice of the caregiver) serve as incentive signals that elicit a dopamine-related anticipatory phase of ‘wanting’. Oxytocin is thought to enhance the perceptual salience of such signals, which facilitates their interpretation and influences affiliative approach behavior. Physiological evidence suggests that oxytocin neurons in the hypothalamus may directly project to the VS, activating dopamine release and influencing locomotor behavior [153,154]. As the affiliative person is reached, the exchange of reward (for example, pleasant touch, approval) triggers opioid release, which promotes a state of pleasure and ‘liking’. During this consummatory phase of ‘liking’, oxytocin has the potential to increase opioid release in the brain by up to 300% [155]. In fact, it has been shown that brain areas that are innervated by dopamine and opioids including the VS, the VTA and the prefrontal cortex, contain a high density of oxytocin receptors, making these regions very receptive to changes in central levels of oxytocin [119,156]. Moreover, human imaging demonstrates that intranasally administered oxytocin acts directly on brain processes by modulating social and reward circuitries [157-163]. As a result of the neurochemical interplay between the oxytocin , dopamine, and opioid systems, social encounters and contextual stimuli associated with these encounters (for example, face of the caregiver) are tagged with positive reward value, an essential prerequisite for socially motivated approach behaviors (for example, orienting towards the caregiver).

Evidence is emerging that the oxytocin system is altered in ASD [164], which might be a contributing factor to atypical reward functioning in affected individuals. Dawson and colleagues [40], drawing upon the work of Insel and colleagues [165], proposed a model suggesting that social reward deficits in ASD could result from alterations in oxytocin activity in the context of social interactions, which negatively influence the dopaminergic reward system, and prevent linking of social stimuli with their proper reward value. In fact, two studies have found lower average levels of blood plasma concentrations of oxytocin in individuals with ASD relative to controls [166,167], a decrease that could stem from inefficient or incomplete conversion of oxytocin from its precursor prohormone [168,169]. Genetic associations between ASD and the oxytocin receptor gene, and with the closely related vasopressin gene, have also been reported [170]. As discussed by Insel *et al*. [165], abnormalities in the oxytocin neural pathway could account for many features of ASD, including early onset, predominance in males, genetic loading, and neuroanatomical abnormalities. In fact, postmortem brain analyses were found to have a specific decrease in oxytocin mRNA in the temporal cortex in ASD, which was associated with hypermethylation [171]. The reduced expression of oxytocin receptors in the temporal lobe of persons with ASD is interesting in light of extensive evidence suggesting that the temporal lobe, including the amygdala, has a special role in social perceptual and reward processes [41,70]. The most promising data suggestive of an oxytocin deficiency related to the development of ASD come from pioneering studies on the therapeutic effects of intravenous and intranasal oxytocin in individuals with this disorder. These initial studies found that oxytocin , relative to placebo, reduces repetitive stereotypic behaviors [172], improves the recognition and memory of social–emotional information [173,174], and increases cooperative behaviors, trust, and attention to socially informative stimuli (for example, eyes) [166]. Considering the role of oxytocin within the ‘wanting’/’liking’ framework, the positive therapeutic effects on social functioning in ASD may be partially mediated by enhanced socially motivated attention and drive (‘wanting’) towards important social encounters and affiliation (for example, social stimuli or interactions [120,146]). The extent to which this enhancement of social responsiveness is indeed a direct consequence of oxytocin-induced dopamine involvement remains to be determined [175,176]. Innovative research is needed to elucidate the interaction between oxytocin and dopamine as well as between oxytocin and opioid systems to guide both theory (for example, neurobiological basis of social motivation deficits in ASD) and specifically tailored therapeutic approaches for manipulating the underlying neural circuitries (see also the Discussion section).

#### ***Synopsis***

In summary, whereas the opioid ‘liking’ system lacks consistent evidence of involvement, both the dopamine-mediated ‘wanting’ system and particularly the oxytocin system are promising candidates for contribution to atypical reward-related behaviors in ASD. Given the close interaction between dopaminergic and oxytocin mechanisms in driving socially motivated behaviors, any imbalance within the physiological interplay of these two mechanisms has the potential to disrupt social ‘wanting’ tendencies such as seeking out of social activities or friendships. Based on the evidence reviewed in this section, it can be hypothesized that the administration of oxytocin as a pharmacological adjunct to social reinforcement learning procedures may help tag social stimuli with incentive value by stimulating social brain and reward circuitries, and thus potentiating social reward-based ‘wanting’ [177]. We elaborate on this idea below.

## **Discussion**

The fundamental aim of this paper was to present a fine-grained analysis of reward responsiveness in ASD embedded in the ‘wanting’/’liking’ framework [7,8,178]. The evaluation across three converging sets of neurobiological data, including functional neuroimaging responses, electrophysiological recordings, and neurochemical measures, revealed disrupted social reward ‘wanting’ capacities in individuals with ASD, most likely caused by dysfunction of the dopaminergic–oxytocinergic ‘wanting’ circuitry, including the VS, amygdala, and ventromedial prefrontal cortex. This is in line with predictions derived from current (and forthcoming) diagnostic criteria concerning the core social phenotype of ASD, which emphasize difficulties with spontaneous self-initiated seeking of social encounters [1]. Reduced social–motivational drive and interest has also been found in infants at genetic risk for ASD, who are later diagnosed with the disorder [179-181]. By contrast, empirical evidence for compromised reward ‘liking’, including social reward, is inconclusive, and at this point, not strongly supported by data on hand [54]. However, the literature is still very new, with few studies, each of which varies in what could be important details of their methodology. Hence, it would be premature to try to draw firm conclusions. Much more research is needed to elucidate the nuances (for example, developmental trajectories, heterogeneity) of reward ‘wanting’ versus ‘liking’ in ASD. Moreover, what still needs to be determined empirically is the extent to which a deficient dopaminergic–oxytocinergic ‘wanting’ circuitry in fact affects motivational actions, social functioning and development in affected individuals.

### **Social motivation theory of ASD**

It should be acknowledged that disrupted social engagement certainly falls short of explaining the full picture of the ASD phenotype, including non-social weaknesses and strengths [2]. However, on the basis of the evidence summarized in this paper and by others [2,40,41,54,56], it seems to be crucial to elucidate the brain–behavior underpinnings of aberrant social motivation, a core deficit in ASD, and its diversity among affected individuals. Such knowledge should advance our understanding of the etiological and phenotypic complexities of the autism spectrum, but may also lead to an understanding of the unique risk and protective factors that can be specifically targeted by intervention and prevention researchers [182]. This will help create tailored treatment programs that can optimize functional outcome and, thus positively influence the developmental course of an affected individual or an individual with a genetic risk for the disorder.

By integrating behavioral observations and biological findings, the social motivation theory of ASD posits that ASD can be construed as an extreme case of reduced social interest. This reduction fundamentally alters how individuals with ASD attend to and interact with the world, depriving them of crucial social perceptual and social cognitive learning opportunities [2,4,40,41,54,183]). The theory assumes that the social motivation impairment is attributable, at least partially, to dysfunction of the dopaminergic–oxytocinergic vmPFC–VS–amygdala brain network [2,40]. Consistent with the expression of the core social phenotype [1] and in line with evidence reviewed in this paper, it is most likely that the lack of social-seeking tendencies in individuals with ASD is caused by an inability of the ‘wanting’ circuit to activate motivational behaviors, particularly in social contexts. We extend this idea by hypothesizing that diminished activation associated with social ‘wanting’ may additionally be affected by exaggerated brain receptivity for non-social objects of high autism salience [60,72,184]. Such circumscribed stimuli may indeed have greater than normal incentive valence for individuals with ASD (for yet unclear reasons), and thus potentially further absorb resources typically dedicated to social interests and attention [184].

An alternative, but not mutually exclusive explanation is that social anxiety, as opposed to diminished social motivation, contributes to social avoidance behaviors in ASD [185]. The unpredictable nature inherent in social encounters might be particularly challenging for individuals with ASD [186], and could promote symptoms of anxiety and withdrawal. In fact, unpredictability has recently been reported to elicit anxiety-like behavior in humans and mice, reflected in amygdala involvement at the brain level [70,187]. Further inquiries are clearly needed to clarify the extent to which social anxiety, relative to low social motivation, is implicated in the core social phenotype of ASD.

### **Implications for treatment**

A weak social ‘wanting’ circuitry suggests that particularly strong social cues, in combination with other strategies to boost attention and engagement [2], might be necessary for individuals with ASD to help them establish motivational salience and initiate desired behaviors [4,42]. The initial behavioral modification programs attempted to create responsiveness to social stimuli through operant conditioning using already powerful reinforcers (for example, food [43,188,189]), but this approach only showed limited generalization effects to daily social function [42]. Later programs focusing on the functional (that is, contextual) analysis of problem behaviors were more successful in addressing core deficits [3,5,6,32,42,190-196]. Not surprisingly, diminished motivational tendencies, in particular reduced self-initiated social encounters, have been identified as a pivotal area of intervention in ASD [42,197]. The literature reviewed in this paper clearly supports this approach by highlighting that impaired social motivation is most likely caused by functional disruptions in the ‘wanting’ circuitry.

A number of intervention-related factors that promote successful treatment outcome in ASD, in terms of social and cognitive gains, have been defined (for example, initiation of intervention before the age of 4 years, intense delivery of h more than 20 hours/week for at least 2 years, incorporation of reinforcement principles; [182]). However, the percentage of affected children who actually profit from behavioral treatment, let alone reach optimal outcome (recovery), differs widely between studies, ranging from 3% to 50% [4]. Most recently, child characteristics that are predictive of response to intervention have been described. Besides intellectual and language abilities, several studies suggest that individual differences in the degree of social motivation tendency are associated with treatment efficacy [182]. Young children with ASD who display stronger social approach (‘wanting’) and fewer avoidance behaviors at treatment onset are more responsive to early intensive behavioral interventions than are passive and avoidant children [198-202]. It seems that children with greater inherent social interest engage more actively and constructively in the intervention procedures, which results in greater benefits. However, Koegel and colleagues [198] found that children with few social-seeking attempts and initially poor treatment outcome were able to learn social self-initiations (for example, seeking adults for help and attention), which then led to favorable intervention effects (for example, having best friends, going to birthday parties and sleepovers, talking to friends on the phone). Similar findings were reported by Kasari *et al*. [203]. Taken together, impaired social motivation, a core deficit with considerable variation among individuals with ASD, is malleable, and its successful treatment contributes to better functional outcome.

If our conclusion about a biobehavioral imbalance of diminished social ‘wanting’ and excessive seeking of ASD-specific stimuli is correct, it argues in favor of capitalizing on the latter to improve the former. This idea is not new, as there have been several small-scale behavioral intervention studies reporting that the use of child-preferred objects and activities to promote social initiations indeed increases this type of behavior in ASD [42,204-206]. Because many children with ASD show circumscribed interests in mechanical systems [207], we foresee that new technologies, including computers (for example, video games), internet platforms, or robots, also have the potential, if used thoughtfully, to become therapeutically meaningful [208,209].

Moreover, given the possible involvement of the dopaminergic–oxytocinergic circuitry in social motivation deficits, oxytocin pharmacotherapy seems to be a promising treatment approach for ASD [120]. Intriguingly, animal models show that oxytocin can exert therapeutic effects via binding to the vasopressin receptor V1a in the absence of functional oxytocin receptors [210]. Therefore, administering oxytocin (for example, in the form of a nasal spray), or other substances that enhance central oxytocin turnover might be effective in ASD even in the presence of a disrupted oxytocin system [164]. However, most recent literature reviews conclude that oxytocin pharmacotherapy alone will probably have only limited long-term beneficial effects on pro-social functioning [120,149,152,164]. Combining oxytocin with a structured social-skills training regimen, as part of ABA [42] or computerized gaming, has the potential to be a more effective treatment for social motivation impairments and other skill deficits in ASD [177,211]. Ultimately, it is hoped that such combination treatments will result in optimal outcome for a larger percentage of individuals with ASD than traditional behavior-only approaches.

### **Obstacles and recommendations for future research**

One significant obstacle in the field concerns the tremendous phenotypic heterogeneity of ASD, with important consequences for intervention outcome. There is shockingly little research on that topic to date. Besides pre-treatment variables such as the degree of social motivation capacities, other subject characteristics have been described that can potentially impair reward sensitivity, and thus may contribute to treatment failure in ASD. For instance, co-morbid traits, such as depression, anxiety, or inattention/hyperactivity, are often associated with ASD [212], and are known to affect general reward processing [213,214] and intervention response [215]. Moreover, research indicates that sleep deficit, a problem commonly exhibited by individuals with ASD [216], alters reward receptivity, particularly to social rewards [217-219]. Lastly, individual differences in temperament and achievement/intrinsic motivation tendencies have been documented in ASD [220,221], and can result in differential responsiveness to reward [222,223]. Taken together, these findings emphasize the need for rigorous assessments and optimal management of such factors to increase the likelihood of optimal outcome. However, laboratory-based measures that accurately quantify reward responsiveness and motivational tendencies in ASD are lacking, and have yet to be developed. Additionally, a goal for future research is to identify neurobiological ‘traits’ for example, [224] and genetic moderators [225,226] that are predictive of treatment response. This will allow determination of which treatment option works well for a specific subset of individuals with ASD but not for others.

A second obstacle relates to methodological shortcomings in experimental research on reward responsiveness. For instance, there is a lack of research into biological rewards such as food items, despite these being a most commonly used reinforcer during intervention. Furthermore, physiological or drive states have been neglected, although intervention researchers have shown that individuals with ASD respond at higher rates to social rewards under conditions of social deprivation than during social satiation, for example [227]. Lastly, many reward stimuli used in experimental tasks lack ecological validity. For instance, static images of smiling people and coins have been used as social and monetary rewards respectively [61,62,92]. Taken together, the field needs an innovative line of experimental research that systematically assesses responsiveness to different types of treatment-relevant reinforcers under different physiological states at both the behavioral and the brain level. In this context, more ecologically valid stimulus sets and experimental paradigms are necessary to fully understand the extent to which there is any domain specificity to the reward dysfunction in ASD, which would be most consistent with the core autism phenotype. Ultimately, enhanced characterization of these processes will contribute to our understanding of the biobehavioral heterogeneity of ASD and allow for the development of better, more personalized treatments.

## **Conclusion**

The intent of this review was to spark interest in this promising area of research and to move discussion of social motivation and reward-based learning more into the mainstream of the autism research community. Given the prominent role that behavioral interventions play in the lives of families affected by ASD, and that these interventions can be ineffective in a large percentage of cases, its seems imperative that new research agendas include the study of reward mechanisms, supported by efforts of both cognitive science and cognitive neuroscience. Firm conclusions about the relative importance of ‘wanting’ and ‘liking’ components of reward in ASD are not yet possible, owing to the paucity of studies. However, the data published to date indicate the importance of social motivation (‘wanting’) and of the forces that propel social striving as crucial to understanding and treating autism. We expect that the next decade of systematic research will elucidate with greater detail the basis of these deficits, and that this knowledge will be translated into more effective treatments.

## **Abbreviations**

ABA = Applied behavior analysis; ACC = Anterior cingulate cortex; ADI-R = Autism diagnostic interview-revised; ADOS-G = Autism Diagnostic Observation Schedule-Generic; ASD = Autism Spectrum Disorder; BOLD = Blood oxygen level-dependent; CSF = Cerebrospinal fluid; CS = Conditioned stimulus; DTI = Diffusion tensor imaging; EEG = Electroencephalography; ERP = Event-related potential; fMRI = Functional magnetic resonance imaging; FRN = Feedback-related negativity; LC-NE = Locus coeruleus norepinephrine; NAcc = Nucleus accumbens; OFC = Orbitofrontal cortex; PET = Positron emission tomography; PDD-NOS = Pervasive developmental disorder not otherwise specified; SPECT = Single photon emission computed tomography; SN = Substantia nigra; TDC = Typically developing children/controls; UCS = Unconditioned stimulus; vmPFC = Ventromedial prefrontal cortex; VS = Ventral striatum; VTA = Ventral tegmental area.

## **Competing interests**

The authors declare that they have no competing interests.

## **Authors’ contributions**

GK drafted the manuscript; GK, CC and RTS contributed to the theoretical approach presented in the manuscript; and VT prepared the illustrations. All authors contributed to writing the manuscript. All authors read and approved the final manuscript.
